# Biomaterials for Drug Delivery and Human Applications

**DOI:** 10.3390/ma17020456

**Published:** 2024-01-18

**Authors:** Paolo Trucillo

**Affiliations:** Department of Chemical, Materials and Industrial Production Engineering, University of Naples Federico II, Piazzale V. Tecchio, 80, 80125 Naples, Italy; paolo.trucillo@unina.it; Tel.: +39-32-9656-6043

**Keywords:** biomaterials, drug-delivery systems, regenerative medicine, diagnostic tools, targeting

## Abstract

Biomaterials embody a groundbreaking paradigm shift in the field of drug delivery and human applications. Their versatility and adaptability have not only enriched therapeutic outcomes but also significantly reduced the burden of adverse effects. This work serves as a comprehensive overview of biomaterials, with a particular emphasis on their pivotal role in drug delivery, classifying them in terms of their biobased, biodegradable, and biocompatible nature, and highlighting their characteristics and advantages. The examination also delves into the extensive array of applications for biomaterials in drug delivery, encompassing diverse medical fields such as cancer therapy, cardiovascular diseases, neurological disorders, and vaccination. This work also explores the actual challenges within this domain, including potential toxicity and the complexity of manufacturing processes. These challenges emphasize the necessity for thorough research and the continuous development of regulatory frameworks. The second aim of this review is to navigate through the compelling terrain of recent advances and prospects in biomaterials, envisioning a healthcare landscape where they empower precise, targeted, and personalized drug delivery. The potential for biomaterials to transform healthcare is staggering, as they promise treatments tailored to individual patient needs, offering hope for improved therapeutic efficacy, fewer side effects, and a brighter future for medical practice.

## 1. Introduction

The advancement of efficient drug-delivery systems is essential for enhancing patient outcomes across a range of medical conditions [1,2]. Clinical consequences can vary significantly based on numerous factors, including the quality of healthcare received and the individual patient’s characteristics, thus influencing clinical decision-making [3]. Medical conditions are incredibly variable, ranging from acute illnesses like infections and injuries to chronic diseases such as diabetes. Consequently, patient outcomes can range from complete recovery to long-term therapies [4,5]. The access to quality healthcare significantly influences patient outcomes. In developed countries, patients generally benefit from better access to high-quality medical care, resulting in improved outcomes. Conversely, resource-limited situations, inadequate healthcare infrastructure, a lack of trained personnel, and limited access to essential medications and treatments may compromise patient outcomes. Additionally, preventive medicine plays a crucial role in reducing patient side effects, through proactive measures like routine screenings, vaccinations, lifestyle modifications, and early disease detection. These measures contribute significantly to improving patient well-being [6,7,8]. Furthermore, patient outcomes are not solely determined by medical interventions, but also by education, housing, and access to healthy food [9]. Innovative materials contribute to advancements in medical technology, offering novel solutions for development and application [10,11,12]. Breakthroughs in electronics, software, and robotics have been crucial in advancing medical technology. However, the development and application of innovative materials play a central role in shaping the future of medicine. This evolution extends beyond previous limits, impacting diagnostic tools, implantable devices, drug-delivery systems, and regenerative medicine. Innovative materials catalyze a revolution in medical technology, marked by a multifaceted approach involving materials science, nanotechnology, and biotechnology. In the context of medical technology, these materials cover a diverse range, each tailored with specific properties to address unique healthcare challenges [13,14,15,16,17]. Among these, polymers emerged as vital players, enabling the creation of biocompatible and bioresorbable structures [18] for medical devices [19,20], tissue engineering scaffolds [21,22,23], and drug-delivery systems [20,21,22,23,24].

Biobased and biodegradable polymers offer versatile engineering possibilities, allowing for controlled degradation rates, tailored mechanical properties, and surface characteristics that harmonize with the human body. Innovative polymer materials have led to the development of bioresorbable stents, transforming cardiology by replacing permanent metallic stents. These advancements reduce long-term complications and eliminate the necessity for additional invasive procedures [25,26,27,28]. Controlled degradation plays a vital role in the functionality of medical implants, particularly those with a temporary purpose, such as stents or scaffolds in tissue engineering. This controlled breakdown aligns with the healing process, preventing complications associated with permanent implants like inflammation or the need for additional removal surgeries. Biodegradable materials, including polymers like polylactic acid (PLA) or polyglycolic acid (PGA), naturally break down into non-toxic byproducts, in stark contrast to non-biodegradable materials like certain metals that can cause long-term complications by remaining in the body indefinitely. Opting for biodegradable products in medical implants not only reduces the risk of chronic inflammation but also eliminates the need for additional removal surgeries, aligning with the objective of enhancing patient outcomes while minimizing overall bodily impact.

Nanomaterials, showcasing remarkable properties at the nanoscale, play a pivotal role in advancing medical technology [22,29,30,31,32]. Their applications span a wide spectrum, from targeted drug delivery [33,34,35,36] to the creation of imaging agents [37,38,39,40,41], and the development of cutting-edge diagnostic tools [42,43]. Quantum dots, for example, offer adjustable fluorescence properties, facilitating more precise and efficient imaging, which is particularly crucial in cancer diagnosis and treatment monitoring [44,45,46]. Simultaneously, the exploration of carbon nanotubes and graphene-based materials is underway, as they hold potential for developing lightweight and robust medical devices with enhanced electrical conductivity, making them ideal for applications like neural interfaces and prosthetics [47,48,49,50]. Biological materials, including biomimetic polymers and biologically derived substances, are contributing significantly to medical technology by mimicking natural tissues and structures, thereby enhancing compatibility with the human body [51,52,53,54]. Innovations in tissue engineering leverage these biomaterials to construct functional organs and tissues, potentially alleviating the demand for organ transplantation and reducing rejection rates. Additionally, bioactive coatings on medical implants, such as artificial joints and dental implants, promote better integration with surrounding tissues and reduce the risk of complications [55,56].

Metamaterials are an emerging category of materials [57,58], distinguished by intentionally engineered properties that generally are not possible to find in nature. They demonstrate promise for medical applications by facilitating the creation of devices endowed with exceptional abilities to manipulate light, sound, and electromagnetic waves. In medical imaging, metamaterial-based lenses and cloaking devices can lead to improved diagnostic tools that provide higher resolution and sensitivity [57,58,59,60,61]. These innovations contribute to early disease detection and personalized treatment planning.

Moreover, innovative materials play a crucial role in the advancement of smart medical devices. Shape memory alloys, for instance, facilitate the fabrication of self-expanding stents that can be remotely triggered to change shape, providing precise control during deployment. This adaptability not only diminishes the invasiveness of surgical procedures but also enhances patient comfort [25,27,62]. Similarly, conductive polymers and flexible substrates are essential for the development of wearable medical devices, like biosensors and electronic skins, which can continuously monitor a patient’s health and transmit real-time data to healthcare professionals, fostering proactive and personalized care.

Innovative materials also play a crucial role in advancing regenerative medicine, a field focused on harnessing the body’s natural healing mechanisms to repair or replace damaged tissues and organs [63,64]. Scaffold materials with intricate microarchitecture guide tissue regeneration, while hydrogels provide a supportive environment for stem cell growth and differentiation. Seeded with cells and bioactive molecules, these biomaterials hold the potential to revolutionize treatments for conditions ranging from spinal cord injuries to degenerative diseases.

The utilization of innovative biomaterials, exemplified by bioactive glasses and glass-ceramics, has opened new avenues in the realm of drug delivery. Many bioactive glasses and glass-ceramics exhibit exceptional biocompatibility and bioactivity, rendering them promising candidates for designing advanced drug-delivery systems. These materials possess the ability to interact harmoniously with the biological environment due to their controlled degradation rates and surface characteristics. In the context of drug delivery, these biomaterials offer a versatile platform, enabling the incorporation of therapeutic agents and facilitating controlled release. The porous structure of bioactive glasses and glass-ceramics enhances drug-loading capacity while promoting localized and sustained drug release, leading to improved treatment outcomes. This innovative approach not only optimizes drug delivery but also harnesses the regenerative properties of bioactive materials, contributing to the overall efficacy and success of medical interventions.

The main goal of the present work is to highlight the critical importance of biomaterials in developing effective drug-delivery systems to enhance patient outcomes across a wide range of medical conditions. Innovative materials are significant in shaping the future of medicine and playing a role in revolutionizing healthcare. This work also discusses a multifaceted approach involving materials science, nanotechnology, and biotechnology. The reference selection criteria are comprehensive, focusing on ensuring relevance and credibility. References align with the main theme of developing effective drug-delivery systems and utilizing innovative materials in medical technology. The review emphasizes biomaterials, polymers, nanomaterials, biomimetic polymers, and metamaterials, exploring their applications in drug delivery, medical devices, regenerative medicine, and smart medical devices. Notably, the review provides a contemporary overview by incorporating recent updates in the field of biomaterials in medical technology. The chosen references cover a wide range of materials science, nanotechnology, and biotechnology, addressing applications such as medical imaging, diagnostic tools, tissue engineering, regenerative medicine, and smart medical devices. The inclusion of references tied to key milestones and breakthroughs enhances the review’s credibility, ensuring a well-supported exploration of topics related to medical technology and innovative materials.

## 2. Biomaterials

Biomaterials, crafted to interact with biological systems, have become a cornerstone in the realm of drug delivery and various human applications. These materials offer a level of customization that allows for the precise control of drug-release kinetics, thus significantly improving bioavailability while enabling the targeting of specific tissues or cells [65]. Biomaterials, by significantly reducing side effects and improving therapeutic effectiveness, have expanded their applications beyond medicine, infiltrating diverse scientific and technological domains [66,67,68]. Their innate properties and incredible versatility have ushered in a transformative era in healthcare, research, and innovation, redefining the standards of patient care and scientific exploration. The crucial importance of biomaterials becomes particularly apparent in the field of drug-delivery systems. Through careful engineering, these materials can be finely customized to accommodate a wide range of therapeutic agents, ensuring the controlled and gradual release of medications over time. This control capability not only enhances the drug’s efficacy but also minimizes adverse reactions and side effects, thereby improving patient comfort and compliance [69,70,71,72]. Furthermore, the specificity of biomaterials allows for the targeted delivery of drugs to specific cells or tissues [73,74,75]. Beyond their involvement in drug delivery, biomaterials showcase their versatility in numerous other medical applications. They act as the fundamental building blocks for a diverse array of medical devices, encompassing artificial joints, dental implants, and scaffolds for tissue engineering [76,77,78]. These materials promote integration with the body, reducing the risk of complications and enhancing the longevity of such devices.

Biomaterials for drug delivery and human applications constitute a fundamental support in advancing therapeutic interventions. To meet the diverse and intricate demands of such applications, several material requirements must be carefully considered. Among these, particle size is a critical parameter, with nanoparticles being commonly favored for drug delivery due to their enhanced bioavailability and targeted delivery capabilities. Additionally, microparticles may find utility in providing sustained drug release. High surface area, a key consideration, influences the drug-loading capacity and interactions with biological components, playing a crucial role in controlled release systems. The biocompatibility of biomaterials is imperative, necessitating materials that are non-toxic and compatible with biological systems to avoid adverse reactions. Biodegradability is often preferred, allowing biomaterials to break down over time, reducing the need for surgical removal. The surface charge and functional groups impact cellular interactions, while mechanical properties, thermal stability, and sterility ensure the suitability of biomaterials for various applications. Efficient drug-loading and controlled release mechanisms, along with adherence to regulatory standards, further contribute to the successful design and implementation of biomaterials for drug delivery and human applications.

In this field, the carriage mechanisms play a fundamental role in ensuring the effective and targeted delivery of therapeutic agents. These mechanisms involve the encapsulation, entrapment, or attachment of drugs within or onto biomaterial carriers. Nanoparticles, liposomes, microparticles, and hydrogels are among the commonly employed carriers. Encapsulation involves enclosing drugs within a carrier system, providing protection and controlled release. Entrapment involves trapping drugs within the carrier matrix, while attachment refers to the coupling of drugs onto the carrier’s surface. These carriage mechanisms serve to enhance drug stability, prolong circulation time, and facilitate specific localization at the target site, minimizing systemic side effects. Tailoring the carriage mechanisms allows for precise control over drug-release kinetics, improving therapeutic efficacy. Furthermore, the design of biomaterial carriers considers factors such as biocompatibility, biodegradability, and the ability to respond to environmental stimuli, ensuring a sophisticated and tailored approach to drug delivery for diverse medical applications.

The influence of biomaterials extends far beyond medical devices, as they play a crucial role in the field of regenerative medicine. Scaffold materials with intricate microarchitecture can guide tissue regeneration, offering a promising avenue for constructing functional organs and tissues [79,80,81,82].

The application of biomaterials is constantly extending its impact across diverse domains, with a notable emphasis in regenerative medicine. Hydrogels, a significant biomaterial component, exhibit remarkable potential by nurturing the growth and differentiation of stem cells, providing promising solutions for conditions like spinal cord injuries and degenerative diseases. In the ever-evolving landscape of scientific research, biomaterials showcase versatility, contributing not only to healthcare but also propelling advancements in biotechnology and nanotechnology.

### 2.1. Drug-Delivery Systems

One of the most significant applications of biomaterials lies in the design of drug-delivery systems [83]. These materials can be designed to encapsulate and release pharmaceutical compounds in a controlled and targeted manner. This approach offers several advantages, including improved drug efficacy, reduced side effects, and prolonged release for chronic conditions. For instance, biodegradable polymer nanoparticles can be loaded with drugs and injected into the body, ensuring a sustained release at the site of action. Biomaterials for drug-delivery systems can include polymers, lipids, and other materials that are used to encapsulate and control the release of drugs. A list of common biomaterials used in drug-delivery systems is provided below (Table 1).

### 2.2. Tissue Engineering

Researchers developed scaffolds made from biocompatible materials like hydrogels, ceramics, and polymers to support cell growth and tissue regeneration. These scaffolds provide a structural framework for cells to attach, proliferate, and differentiate, ultimately leading to the creation of functional replacement tissues and organs such as bones [94,95,96]. Tissue engineering involves the use of biomaterials to create functional tissues for regenerative medicine and transplantation. A list of common biomaterials used in tissue engineering and their applications is proposed in Table 2.

### 2.3. Implantable Devices

The development of implantable medical devices, such as artificial joints, pacemakers, and stents, heavily relies on biomaterials. These materials must be biocompatible to ensure they integrate seamlessly with the body’s tissues and do not trigger an immune response. Biomaterials such as titanium alloys and biodegradable polymers have been instrumental in enhancing the quality of life for patients in need of such devices. Materials enlisted in Table 3 should be simultaneously biocompatible, durable, while performing specific functions. A list of common biomaterials used in implantable devices and their applications is provided below (Table 3).

### 2.4. Diagnostic Tools

Biomaterials are also essential in the development of diagnostic tools. Nanoparticles coated with specific biomolecules can be used to detect diseases at an early stage. For example, magnetic nanoparticles functionalized with antibodies can bind to cancer cells, allowing for their magnetic separation and subsequent identification. A list of common biomaterials employed in the fabrication of diagnostic tools is provided below (Table 4).

Table 4 provides a general overview of some biomaterials commonly used in diagnostic tools, and there are many other specialized materials and combinations used for specific applications within the field of diagnostics.

### 2.5. Regenerative Medicine

Researchers have unlocked the intriguing potential of biomaterials within the field of regenerative medicine. Notably, stem cell therapy frequently utilizes scaffolds and matrices crafted from biomaterials to direct the transformation of stem cells into specific cell types. This presents a hopeful prospect for addressing ailments such as spinal cord injuries, neurodegenerative diseases, and heart damage. In Table 5, a list of commonly utilized biomaterials in regenerative medicine is reported.

### 2.6. Drug Screening and Research

Several innovative biomaterials are being employed to generate three-dimensional cell cultures, commonly referred to as organoids or spheroids. These systems mimic the in vivo environment more closely than traditional two-dimensional cell cultures, allowing for more accurate drug screening and testing. Table 6 contains a list of specific biomaterials commonly used in drug screening and research, along with their applications and advantages.

### 2.7. Bioimaging

Biomaterials are also used as contrast agents in various imaging techniques, such as magnetic resonance imaging (MRI) and ultrasound. These materials enhance the visibility of specific tissues or structures, aiding in the diagnosis and monitoring of diseases. Table 7 reports a list of specific biomaterials commonly used in bioimaging, along with their applications and advantages.

### 2.8. Vaccine Development

Biomaterials are playing a crucial role in vaccine development, particularly in the context of mRNA vaccines like those developed for COVID-19. Lipid nanoparticles serve as carriers for the fragile mRNA molecules, protecting them and facilitating their delivery into cells to trigger an immune response. A list of specific biomaterials commonly used in vaccine development is provided below (Table 8).

## 3. Synthetic, Biobased, and Biodegradable Biomaterials

Derived from renewable biological sources like plants, animals, or microorganisms, biobased materials are environmentally friendly substances. Among these, biodegradable materials have a unique ability to naturally decompose or transform into simpler compounds over a short period, facilitated by microorganisms like bacteria and fungi. This subset of biomaterials plays a crucial role in medical science and material engineering, where categorization based on degradability is fundamental. Biomaterials are broadly classified into two categories: biodegradable and non-biodegradable. Biodegradable materials, including magnesium-based alloys and natural biopolymers like chitosan and collagen, break down over time, allowing natural absorption or excretion by the body. For instance, magnesium-based alloys are renowned for their orthopedic implants and underscore the importance of biodegradability in minimizing long-term impact. In contrast, non-biodegradable materials, such as certain metals and synthetic polymers, maintain structural integrity for extended periods, crucial for applications demanding long-term stability in medical devices. Additionally, biobased materials, or biomass-based materials, are derived from renewable biological sources and can be further categorized based on their biodegradability and compatibility with biological systems. These materials, which include biodegradable substances capable of breaking down into non-toxic compounds, water, and carbon dioxide, are intended for safe use within the human body or other biological environments, minimizing adverse effects [194]. These materials are intended to be safe for use within the human body or other biological environments without causing toxicity, inflammation, rejection, or other negative effects.

Among biodegradable polymers [195], polybutylene succinate (PBS) is a biobased polymer derived from succinic acid and 1,4-butanediol, which can be obtained from renewable sources like corn. It is biodegradable and used in packaging and agricultural films; moreover, polyglycolic acid (PGA) is a biobased and biodegradable polymer used in medical sutures and other biomedical applications; Poly-3-hydroxybutyrate-co-3-hydroxyvalerate (PHBV) is a biodegradable copolymer produced by certain bacteria and can be used in various applications, including packaging and medical devices; starch blends, with other biodegradable polymers, can result in materials that are both biobased and biodegradable. These blends are used in items like biodegradable bags and packaging; certain forms of polyethylene glycol (PEG) are biodegradable. They are used in various medical and pharmaceutical applications.

Concerning biobased and non-biodegradable polymers [196], polylactic acid (PLA) is obtained from fermented plant starch, usually corn. While it is often considered biodegradable under industrial composting conditions, it may not readily biodegrade in natural environments; moreover, polyethylene (PE) is a biobased and non-biodegradable polymer made from sugarcane: some companies produce a type of polyethylene using biobased ethylene derived from sugarcane ethanol. While this reduces the carbon footprint, it does not necessarily make the polymer biodegradable; polyethylene terephthalate (PET) is a biobased and non-biodegradable polymer made from plant sources: plant-based PET is derived from biobased ethylene glycol made from renewable sources like sugarcane. However, the resulting PET is not biodegradable; polyamides (Nylon) is a biobased and non-biodegradable polymer made from castor oil: some biobased polyamides are produced using castor oil, which is a renewable resource. These materials can have improved sustainability but are generally not biodegradable.

A non-biobased and biodegradable polymer [197] is polybutylene adipate terephthalate (PBAT), which is commonly used in compostable plastic products; polycaprolactone (PCL) is a non-biobased, biodegradable polymer used in various applications, including drug-delivery systems and 3D printing; polyvinyl alcohol (PVA) is a synthetic polymer that can be made biodegradable and is used in applications like water-soluble packaging films; polyethylene oxide (PEO) is a synthetic polymer that is water-soluble and biodegradable under certain conditions.

The last member of this classification is polyethylene (PE), a non-biodegradable polymer [198,199]. It is used in various applications, including plastic bags and containers; polypropylene (PP) is another common non-biodegradable polymer used in products such as packaging, automotive parts, and textiles; polyvinyl chloride (PVC) is a versatile synthetic polymer but is not biodegradable. It is used in construction materials, pipes, and vinyl products; polystyrene (PS) is non-biodegradable and is used in disposable cutlery, packaging materials, and foam products; polyethylene terephthalate (PET) is widely used for plastic bottles and containers but is not biodegradable; polyurethane (PU) is a non-biodegradable polymer used in a wide range of applications, including foam insulation and flexible foam products; acrylonitrile butadiene styrene (ABS) is a non-biodegradable polymer commonly used in 3D printing and automotive components; polycarbonate (PC) is used in eyeglass lenses, CDs/DVDs, and various industrial applications and is not biodegradable.

A diverse range of biomaterials have a fundamental role in medicine and biomedical engineering, each selected for specific applications based on their unique properties. Polymeric biomaterials, like PE, PU, PLGA, and PEG, are widely used in medical devices and drug delivery. Metals such as titanium and stainless steel are preferred for orthopedic and dental implants, while ceramics like hydroxyapatite find use in similar scenarios. Natural polymers such as collagen and chitosan are integral to tissue engineering, and biodegradable polymers like PLA, PGA, and PCL are essential in drug delivery and tissue engineering. Hydrogels, including polyacrylamide and polyethylene glycol diacrylate (PEGDA), play pivotal roles in both drug delivery and tissue engineering. Composite biomaterials, like carbon fiber-reinforced polymers, offer versatile solutions to complex biomedical challenges.

Cutting-edge biomaterials revolutionize diverse medical applications, providing solutions for bioresorbable needs through engineered alternatives like bioresorbable magnesium alloys. Biomimetic materials, inspired by natural tissues, play a crucial role in tissue engineering, with examples including silk-based biomaterials and extracellular matrix (ECM) analogs. Nanomaterials, such as gold nanoparticles, carbon nanotubes, and nanofiber scaffolds, offer precision in drug delivery, imaging, and tissue engineering at the nanoscale. Some biomaterials sourced from biology involve decellularized tissues, xenografts (e.g., pig heart valves), and autografts (e.g., a patient’s tissue). Synthetic biodegradable polymers like PLA, PGA, and PCL ensure controlled degradation, vital for gradual medical applications. This diverse array of biomaterials showcases the forefront of medical science, delivering innovative solutions for a wide range of healthcare challenges. In drug delivery, biomaterials take various forms—polymers, lipids, nanoparticles, hydrogels—each selected for unique advantages in line with therapeutic goals and drug properties.

### 3.1. Synthetic and Natural Polymers

Synthetic and natural polymers, such as polyethylene glycol (PEG), polylactic acid (PLA), and chitosan, play a crucial role in drug delivery by providing a versatile platform for controlled release and encapsulation. PEG’s hydrophilicity and biocompatibility allow precise customization for diverse therapeutic agents, ensuring distinct release kinetics. PLA, a biodegradable synthetic polymer, enables sustained drug release with controlled degradation rates, reducing administration frequency and minimizing side effects. Chitosan, a natural polymer derived from chitin, offers a biodegradable alternative with mucoadhesive properties, facilitating controlled drug release and absorption across biological barriers. The tunable properties of these polymers are paramount in optimizing drug-release profiles, delivering therapeutic agents effectively while minimizing side effects. This capability is essential for various applications, from managing chronic pain with gradual painkiller release to targeted anticancer drug delivery. Additionally, these polymers address challenges related to patient compliance by reducing dosing frequency and enhancing adherence, particularly in chronic conditions where frequent dosing may be burdensome. Therefore, both synthetic and natural polymers have redefined the landscape of drug delivery by offering tunable properties that enable precise control over drug encapsulation and release. PEG, PLA, and chitosan exemplify this adaptability, each with unique characteristics that cater to a wide range of therapeutic needs. The ability to customize drug-release profiles has revolutionized patient care by improving treatment efficacy, reducing side effects, and enhancing patient compliance. As pharmaceutical science continues to evolve, polymers will undoubtedly remain at the forefront of innovation, providing versatile solutions to complex drug delivery challenges and advancing the field of medicine.

### 3.2. Lipid-Based Nanocarriers

The role of lipids in drug delivery has evolved significantly, with lipid-based nanocarriers like liposomes and lipid nanoparticles becoming integral tools in the pharmaceutical industry [200]. These carriers are specifically designed to encapsulate hydrophobic drugs, offering solutions to the persistent challenge of enhancing the solubility and stability of advanced therapeutic agents.

Liposomes, which consist of a lipid bilayer surrounding an aqueous core, have gained prominence as versatile drug-delivery vehicles [201]. The lipid bilayer mimics the structure of biological membranes, making liposomes inherently biocompatible. This biocompatibility reduces the likelihood of adverse reactions, making liposomes a safe choice for drug encapsulation. Moreover, the lipid composition can be tailored to achieve the desired drug-release profile, further highlighting their versatility. By incorporating hydrophobic drugs into the lipid bilayer, liposomes can increase the solubility of these compounds in aqueous environments. This is a crucial advantage, as many drugs with potent therapeutic properties are inherently hydrophobic, posing challenges for their formulation and administration. Liposomes not only enhance drug solubility but also protect the drug from degradation, extending its shelf life and ensuring that the therapeutic payload remains intact until it reaches its intended target within the body.

Lipid nanoparticles represent another powerful lipid-based nanocarrier option. They are typically composed of lipids and are smaller in size compared to liposomes, enhancing drug-loading capacity in some specific circumstances, for example when dealing with highly potent yet hydrophobic drugs [202]. Notably stable, lipid nanoparticles are suitable for prolonged storage and distribution. A key advantage lies in their ability to enable controlled drug release for hydrophobic drugs, achieved by manipulating lipid composition to fine-tune release kinetics. This feature is crucial for maintaining specific drug concentrations in the bloodstream for successful treatment. The modification of lipid surface properties further allows for targeted drug delivery, enhancing precision and efficiency while minimizing off-target effects through functionalization with ligands or antibodies. The transformative impact of lipid-based nanocarriers spans various medical fields. In oncology, liposomes and lipid nanoparticles enhance chemotherapeutic agent delivery, increasing solubility, reducing systemic toxicity, and improving selectivity for cancer cells. These carriers play a pivotal role in effectively delivering hydrophobic antimicrobial agents in infectious disease treatment. Additionally, they find application in encapsulating and delivering genes and nucleic acid-based therapies, addressing unique challenges associated with these emerging treatments [203,204,205].

Lipid-based nanocarriers, such as liposomes and lipid nanoparticles, represent a remarkable achievement in the field of drug delivery. They have revolutionized the administration of hydrophobic drugs, improving their solubility, stability, and therapeutic efficacy. The versatility, biocompatibility, and tunable properties of these carriers make them invaluable tools for the pharmaceutical industry. As research in drug delivery continues to advance, lipid-based nanocarriers will undoubtedly remain at the forefront, offering innovative solutions to improve the treatment of various diseases and enhance the overall patient experience [206].

### 3.3. Nanoparticles

Polymeric nanoparticles can be fabricated starting from biocompatible polymers and have emerged as a cornerstone of modern drug delivery. Their versatility and adaptability make them ideal candidates for encapsulating therapeutic agents, enabling precise drug delivery to specific tissues or cells [207]. One of the most remarkable features of polymeric nanoparticles is their ability to accommodate a diverse range of drugs, from hydrophobic compounds to hydrophilic molecules. By selecting the appropriate polymer and formulation, researchers can tailor the nanoparticles to accommodate the specific physicochemical properties of the drug. This versatility is crucial in addressing the individualized needs of various pharmaceutical compounds and therapeutic scenarios.

Moreover, polymeric nanoparticles offer controlled drug release, allowing for the gradual and sustained release of the encapsulated drug [208]. This controlled release mechanism is essential for maintaining therapeutic concentrations of the drug over an extended period, thereby enhancing its efficacy while reducing potential side effects. The ability to fine-tune the release profile through polymer selection and nanoparticle design has opened new horizons for personalized medicine, ensuring that treatment regimens are tailored to the unique requirements of patients. Beyond drug delivery, polymeric nanoparticles serve as valuable tools in medical imaging. By loading these nanoparticles with imaging agents, they become potent contrast agents in various imaging modalities such as magnetic resonance imaging (MRI) and fluorescence imaging. The incorporation of fluorescent dyes or other contrast agents into the nanoparticles enhances the precision and sensitivity of these imaging techniques. This is particularly significant in disease diagnosis and monitoring, as it allows healthcare professionals to visualize specific biological structures or pathological conditions with unprecedented clarity, often in their earliest stages.

Inorganic nanoparticles, such as gold and silver nanoparticles, bring remarkable capabilities to medical applications. Gold nanoparticles, known for surface plasmon resonance, enhance medical imaging through exceptional light absorption and scattering tunability. Used as contrast agents in computed tomography (CT) scans, they provide high-resolution imaging, crucial for detailed anatomical and pathological insights. Gold nanoparticles also excel in targeted drug delivery by functionalizing surfaces with ligands or antibodies, ensuring precise delivery and maximizing therapeutic efficacy while minimizing damage to healthy tissue. This dual role has propelled theranostics, promising personalized medicine at the intersection of therapy and diagnostics. Silver nanoparticles, with intrinsic antimicrobial properties, find applications in wound care, creams, and dressings, controlling infections through controlled silver ion release. Their adaptability in serving as both targeted drug-delivery vehicles and infection control agents showcases their versatility in medicine. Nanoparticles, whether polymeric for customizable drug delivery or inorganic like gold and silver for advanced imaging and therapy, mark a new era in precision medicine. Their biocompatibility, tunability, and versatility offer solutions to diverse medical challenges, paving the way for more effective treatments, early disease detection, and personalized patient care.

### 3.4. Hydrogels

Hydrogels are a remarkable class of materials that have carved a niche for themselves in the fields of drug delivery and tissue engineering. These three-dimensional networks of hydrophilic polymers, typically water-swollen, provide an ideal environment for drug release and serve as versatile scaffolds in the burgeoning world of regenerative medicine. Hydrogels’ unique characteristics, including biocompatibility, tunable properties, and controlled drug-release capabilities, have positioned them as a central player in revolutionizing how we administer drugs and repair or regenerate damaged tissues. Biocompatibility is a cornerstone of hydrogels, making them particularly well-suited for a wide array of biomedical applications. Their high-water content closely mimics the aqueous environment of living organisms, minimizing adverse reactions and inflammation when introduced into the body. This biocompatibility ensures that hydrogels can be used safely for prolonged periods, crucial for applications in tissue engineering and sustained drug delivery [86,87].

Hydrogels, with their tunable properties, play a pivotal role in revolutionizing drug delivery and tissue engineering. The ability to precisely adjust their chemical composition and physical characteristics makes hydrogels adaptable to diverse applications. In drug delivery, hydrogels serve as a sophisticated platform for controlled drug release, enabling the customization of release profiles for different therapeutic agents. This controlled release is vital for managing chronic conditions, enhancing patient adherence, and minimizing side effects. Hydrogel-based drug-delivery systems can be engineered for single doses or sustained release over extended periods. Their biocompatibility and controlled release properties find applications in targeted drug delivery, such as releasing anticancer agents directly into tumor tissues, reducing systemic exposure, and improving treatment efficacy with fewer side effects. In tissue engineering, hydrogels act as indispensable scaffolds for tissue regeneration, mimicking the natural extracellular environment and facilitating the development of functional replacement tissues. Researchers have leveraged hydrogels to engineer various tissues, from cartilage and bone to skin, and even organs like the liver and heart. This has transformative implications for organ transplants, offering the promise of personalized, lab-grown organs and tissues to address the shortage of donor organs and reduce the risks of rejection. Hydrogels, with their biocompatibility, tunable properties, and controlled drug-release capabilities, represent a significant leap forward in biomedical applications. They continue to shape the future of healthcare by providing novel solutions to complex medical problems, promising more effective and personalized medical treatments through ongoing research and innovation [94,95,96].

## 4. Design Principles for Biomaterial-Based Drug-Delivery Systems

The design of biomaterial-based drug-delivery systems requires a multidisciplinary approach, considering several key principles [27,131,189,194,209,210,211,212,213,214,215,216,217,218].

### 4.1. Biocompatibility

Biomaterials play a vital role in medicine, serving as medical devices, drug-delivery carriers, tissue scaffolds, or imaging agents. Biocompatibility is a fundamental requirement for biomaterials, ensuring their ability to interact with biological systems without causing adverse reactions of toxicity. Biocompatibility is crucial as incompatible materials can trigger immune responses, inflammation, and complications [219]. Avoiding immune reactions is essential to prevent complications, implant failure, or rejection. Rigorous testing, including in vitro and in vivo assessments, is necessary to ensure biomaterials’ biocompatibility and safety for medical applications. Understanding these interactions is pivotal for designing biomaterials that effectively coexist with biological environments.

Biomaterials must be designed with molecules and structures that the body can recognize or metabolize, reducing the risk of adverse reactions [220]. Synthetic polymers, metals, ceramics, and natural polymers each have unique chemical properties that can affect their biocompatibility. Surface properties are equally significant. The surface of a biomaterial can directly influence how it interacts with the biological environment. Surface modification techniques, such as coatings or functionalization, are often employed to enhance biocompatibility by promoting cell adhesion and reducing immune recognition. Moreover, the physical properties of biomaterials, such as their mechanical strength, flexibility, and degradation rate, can also impact biocompatibility. These properties must align with the specific application of the biomaterial. For instance, orthopedic implants need to possess adequate mechanical strength to support the body’s weight, while biodegradable polymers used in tissue engineering should degrade at a controlled rate without causing harm.

In recent years, advancements in biomaterial science have led to the development of smart biomaterials that respond dynamically to the surrounding biological environment, further enhancing biocompatibility. These materials can release drugs, grow with tissues, or adapt their properties to accommodate changes in the body, reducing the likelihood of adverse reactions and improving patient outcomes. In conclusion, biocompatibility is a fundamental and non-negotiable aspect of biomaterial design and development. Ensuring that biomaterials do not induce adverse reactions when interacting with biological systems is essential for the success of medical procedures, devices, and therapies. Biocompatibility testing and the careful consideration of chemical, surface, and physical properties are integral to creating biomaterials that seamlessly integrate with the human body, ultimately advancing the field of medicine and improving the quality of patient care.

### 4.2. Drug-Loading and Release Kinetics

Control over drug-loading and release rates is essential to optimize therapeutic efficacy. In the field of biomaterials, one of the main objectives is to harness precise control over the loading and release of therapeutic agents [221]. This meticulous control is imperative to optimize the therapeutic efficacy of drug-delivery systems. The ability to govern drug-loading and release rates is a fundamental concept that underpins the design and development of biomaterials, enabling tailored, patient-specific treatment regimens and minimizing the potential for adverse effects. Synthetic polymers, natural polymers, liposomes, and nanoparticles are among the commonly employed biomaterials, each offering unique advantages for drug loading. The loading capacity of a biomaterial is dictated by its physical and chemical properties, such as pore size, surface area, and affinity for the drug of interest. Precise control over these characteristics allows researchers to fine-tune the amount of the drug that can be accommodated. This is particularly significant when dealing with drugs with varying potencies or therapeutic ranges, as it enables the customization of drug loading to match the specific needs of the patient or medical condition. Tailored drug loading is not only vital for efficacy but also helps to minimize the potential for overmedication or underdosing, thereby reducing the risk of adverse reactions or treatment failure.

Control over drug-release rates is the complementary facet of this concept, ensuring that the drug is administered in a manner that maximizes its therapeutic benefits. The rate at which a drug is released from the biomaterial carrier directly influences its pharmacokinetics—how it is absorbed, distributed, metabolized, and excreted within the body. The controlled release of a drug ensures that it maintains a therapeutic concentration within the bloodstream, allowing for consistent and sustained therapeutic effects. For drugs that require prolonged action or need to be administered at specific intervals, controlling the release rate is critical. Biomaterials can be engineered to facilitate drug release over varying timescales, from immediate release to extended release over days, weeks, or even months. By manipulating factors such as the biomaterial’s composition, porosity, and degradation rate, researchers can fine-tune the drug-release kinetics to meet the unique requirements of the drug and the patient’s medical condition.

In situations where the therapeutic agent has a narrow therapeutic index or exhibits dose-dependent effects, controlling the release rate becomes even more crucial. For example, in the treatment of certain chronic conditions like diabetes or chronic pain management, maintaining a steady, controlled release of medication is essential to avoid peaks and troughs in drug concentration that can lead to unwanted side effects or inadequate symptom control. This concept of controlling drug-loading and release rates finds applications in a wide array of clinical scenarios. In cancer therapy, for instance, drug-loaded nanoparticles with tunable release rates can be used to precisely deliver chemotherapeutic agents to tumor sites, minimizing collateral damage to healthy tissues and maximizing the therapeutic effect on cancer cells. In the realm of pain management, opioid medications can be delivered using controlled release formulations, reducing the risk of addiction and overdose.

The ability to customize drug-delivery profiles for individual patients is at the forefront of innovation. It allows healthcare providers to optimize treatment regimens based on a patient’s unique physiological characteristics and medical history, ultimately improving the efficacy and safety of drug therapy. Concerning biomaterials, control over drug-loading and release rates is an indispensable concept that forms the cornerstone of drug-delivery systems. It enables the precise customization of drug administration to match the specific needs of patients and medical conditions. This tailored approach not only enhances therapeutic efficacy but also minimizes the potential for adverse reactions, advancing the field of medicine and improving patient outcomes. The capability to harness this control is instrumental in the development of innovative drug-delivery technologies and personalized treatment strategies that hold great promise for the future of healthcare.

### 4.3. Targeting and Specificity

Biomaterials can be engineered to target specific cells, tissues, or organs, reducing off-target effects. Biomaterials have ushered in a new era of precision in medicine, offering the remarkable capability to be engineered with specificity, enabling them to target specific cells, tissues, or organs within the human body. This concept of targeted drug delivery through biomaterials has far-reaching implications, as it not only enhances the therapeutic efficacy of treatments but also minimizes off-target effects, ushering in a paradigm shift in the way we approach medical interventions [222,223].

The ability to target specific cells, tissues, or organs is a pivotal advancement in drug-delivery systems. Traditional methods of drug administration often rely on systemic delivery, where therapeutic agents circulate throughout the entire body, potentially affecting healthy tissues and organs, leading to adverse side effects. This broad exposure can limit the dosage and effectiveness of drugs, posing significant challenges to treatment success. However, biomaterials offer a precision-focused approach. Through careful engineering, they can be designed to recognize and interact with specific molecular markers, receptors, or cellular components that are uniquely present on the target cells or tissues. These engineered biomaterials act as vehicles, shuttling therapeutic agents directly to the intended site of action, thus concentrating the drug’s effects where it is needed most. This specificity is achieved through various strategies, such as surface functionalization with ligands, antibodies, or peptides that bind selectively to the target cells, enabling controlled drug delivery directly to the disease site.

Cancer therapy is a prime example of how biomaterials are transforming the landscape of targeted drug delivery. In oncology, the challenge lies in selectively targeting cancer cells while sparing healthy tissues. Biomaterials, especially nanoparticles and liposomes, have been engineered to carry chemotherapeutic agents directly to cancer cells. This focused approach not only maximizes the concentration of the drug at the tumor site but also minimizes the exposure of healthy tissues to toxic chemotherapy drugs, reducing debilitating side effects such as nausea, hair loss, and immune suppression. Moreover, biomaterials can be used to create drug-eluting implants or devices designed to target specific organs or tissues. For instance, drug-eluting stents coated with specialized biomaterials have been developed to release medication directly into coronary arteries, reducing the risk of restenosis after angioplasty. Similarly, intravitreal implants release drugs directly into the eye to treat retinal diseases, circumventing systemic exposure and associated side effects.

In addition to targeted drug delivery, biomaterials have found applications in regenerative medicine. Tissue engineering often relies on scaffolds made from biocompatible materials, which can be engineered to mimic the structure and properties of the target tissue. These biomaterial scaffolds facilitate the adhesion, proliferation, and differentiation of cells, ultimately promoting tissue regeneration. Whether it is engineering artificial skin, repairing damaged cartilage, or growing functional organs, biomaterials serve as the architectural foundation for these groundbreaking advancements.

The concept of biomaterials for targeted drug delivery extends to gene therapy as well [224,225]. Here, biomaterials can be designed to transport therapeutic genes to specific cell types, addressing genetic disorders or promoting tissue repair. By harnessing the precision of biomaterials, gene therapies can be directed to the precise locations where they are needed, thus maximizing therapeutic potential while minimizing off-target effects. The potential of biomaterials for targeted drug delivery and tissue engineering is not limited to just pharmaceuticals and medical devices. They are increasingly being explored for the delivery of nucleic acid-based therapies, such as RNA and DNA, which hold immense promise in treating genetic diseases and a wide range of disorders. By delivering these therapies directly to the cells or tissues of interest, biomaterials enable the precise and effective correction of genetic abnormalities while minimizing systemic exposure and reducing the risk of unintended consequences.

The ability to engineer biomaterials for targeted drug delivery to specific cells, tissues, or organs represents a monumental shift in modern medicine. This precision-focused approach enhances therapeutic efficacy while simultaneously mitigating off-target effects, ultimately improving patient outcomes, and revolutionizing the way we conceptualize and practice medical interventions. The continued innovation in biomaterial design and engineering promises even greater strides in the development of highly targeted, personalized treatments for a wide range of diseases and conditions.

### 4.4. Stability and Degradation

The concept of designing biomaterials that strike a delicate balance between stability, controlled drug release, and eventual harmlessness within the body is of paramount importance in the fields of drug delivery and tissue engineering. Biomaterials serve as the critical bridge between therapeutic agents and their intended sites of action, and achieving this equilibrium is essential to ensure the safety and effectiveness of medical interventions [226,227]. They must maintain stability while releasing drugs, as this stability ensures the integrity of the drug-delivery system and the controlled release of therapeutic agents. Stability in this context implies that the biomaterial should preserve its structural and chemical properties throughout the period of drug delivery. This is particularly vital for ensuring that the drug remains encapsulated within the biomaterial until it reaches the target site. Stability safeguards the drug against premature release, preventing any sudden surges in drug concentration that could lead to adverse effects or treatment failure. The maintenance of stability in biomaterials involves careful consideration of the material’s physical and chemical properties. Factors such as mechanical strength, degradation rate, and porosity are critical in determining how stable the biomaterial remains during drug delivery. For example, in the case of biodegradable polymers like PLA or PGA, the rate at which the polymer degrades should be finely tuned to match the desired release kinetics of the drug. The degradation of the biomaterial is often a controlled process, ensuring it remains intact until the drug has been delivered.

Controlled drug release, another key aspect of this concept, is fundamental to the effectiveness of drug-delivery systems. The biomaterial should be designed to release the drug in a predictable and sustained manner, consistent with the needs of the patient and the therapeutic agent. Achieving this control involves selecting the appropriate biomaterial and tailoring its properties to the specific drug and medical condition. Drug-release kinetics can be modulated through several mechanisms, including diffusion, erosion, and osmosis. By manipulating these factors, researchers can fine-tune the release profile of the drug, ensuring it meets the therapeutic requirements. Whether it is a drug-eluting stent that steadily releases medication over time to prevent restenosis or a tissue-engineered scaffold that promotes the gradual regeneration of damaged tissues, controlled drug release is crucial for optimizing treatment outcomes.

The third dimension of this concept involves the biomaterial’s eventual degradation in the body. In many cases, biomaterials are engineered to be biodegradable, meaning they are designed to break down harmlessly over time into non-toxic byproducts that can be metabolized or excreted by the body [228,229]. This degradation process is intricately linked to the drug-release kinetics. As the biomaterial degrades, it releases the drug, ensuring that the release profile is consistent with the biomaterial’s breakdown. Biodegradability is particularly advantageous in scenarios where the implanted biomaterial is no longer needed once the drug delivery is complete. For example, in orthopedics, biodegradable bone-fixation devices can be used to hold fractured bones in place during the healing process. As the bone heals, the biodegradable implant gradually breaks down, eliminating the need for a second surgical procedure to remove the device. The harmlessness of the biomaterial’s degradation products is of paramount importance. These byproducts should not cause harm to the body or induce adverse effects. Biodegradable biomaterials are designed to degrade into non-toxic compounds that can be easily excreted or metabolized. This ensures that the biomaterial’s presence in the body is temporary and does not lead to chronic inflammation or other complications.

The concept of designing biomaterials that maintain stability while releasing drugs and eventually degrade harmlessly in the body represents a fundamental pillar of modern drug delivery and tissue engineering. Striking the right balance between stability and controlled drug release is essential to ensure the drug’s effectiveness and safety, while engineering biomaterials to degrade harmlessly minimizes the need for additional medical interventions to remove implanted devices. This careful orchestration of biomaterial properties not only enhances patient outcomes but also lays the foundation for innovative and patient-friendly medical interventions. The continued development and refinement of biomaterials in this context hold great promise for the future of healthcare.

### 4.5. Immunogenicity

The significance of minimizing the immune response to biomaterials is highlighted by immunogenicity. There is a concern about the body’s immune response, which can result in adverse reactions, inflammatory responses, and rejection [230,231,232,233]. This concern is particularly crucial in biomedical applications, including the development of medical devices, implants, and drug-delivery systems. The primary goal is to minimize immunogenicity to ensure the safety, effectiveness, and long-term success of these interventions.. Biomaterials used in medical applications can often be perceived as foreign entities by the immune system, triggering a cascade of events that may result in inflammation, foreign body responses, and, in severe cases, rejection. The immune response can be provoked by a variety of factors, including the biomaterial’s chemical composition, surface properties, and its interaction with immune cells. To mitigate these effects, researchers and biomedical engineers are dedicated to designing biomaterials that minimize their immunogenicity.

One primary focus of minimizing immunogenicity is the selection of biocompatible biomaterials. These are materials that the body is less likely to recognize as foreign or harmful. Materials such as biodegradable polymers, medical-grade metals, ceramics, and certain natural polymers are chosen for their ability to interact harmoniously with the biological environment, reducing the likelihood of an immune response. In contrast, non-biocompatible materials or those with surface characteristics that trigger an immune reaction may result in adverse events and complications. Surface modification is another key strategy in reducing immunogenicity. By altering the surface properties of biomaterials, such as using coatings, functionalization, or surface treatments, researchers can make the material more ‘invisible’ to the immune system. This decreases the chances of the material triggering an inflammatory response or foreign body reaction. Surface modification can also facilitate interactions with specific cells or tissues while avoiding immune recognition.

In the context of implantable medical devices and artificial organs, the design and engineering of biomaterials play a crucial role in minimizing immunogenicity. For instance, cardiac pacemakers and stents made from biocompatible materials like titanium or medical-grade stainless steel minimize the risk of an immune response. Additionally, the development of coatings and surface modifications for such devices helps to further reduce immunogenicity, enhancing their compatibility with the body. Drug-delivery systems also benefit from strategies that minimize immunogenicity. For instance, liposomes and nanoparticles used for drug encapsulation are designed to have surfaces that are less likely to provoke an immune response. These engineered drug carriers are intended to transport therapeutic agents without initiating an inflammatory reaction or being targeted by the immune system, ultimately improving drug-delivery efficiency, and reducing the risk of adverse effects.

In tissue engineering and regenerative medicine, where biomaterials are used to create scaffolds for cell growth and tissue repair, minimizing immunogenicity is vital. These scaffolds should not only provide a suitable physical environment for tissue regeneration but should also avoid immune reactions. To achieve this, biomaterials are carefully selected and engineered to be biocompatible, thus minimizing the likelihood of immunogenic responses and promoting successful tissue integration. Furthermore, in the development of drug and gene therapies, the design of biomaterial carriers is instrumental in reducing immunogenicity. The biomaterials used for these therapies are chosen for their biocompatibility and designed to protect the therapeutic agents from the immune system, allowing for precise drug delivery or gene therapy without undesirable immune reactions.

Minimizing the immune response to biomaterials is essential to prevent adverse reactions and ensure the safety and effectiveness of medical interventions. Through the careful selection of biocompatible materials and the engineering of surface properties to reduce recognition by the immune system, researchers are continuously working to advance the field of biomaterials, making them more compatible with the body and thus contributing to safer and more successful medical treatments. This focus on immunogenicity represents a pivotal step in the ongoing evolution of biomedical technology and patient care.

Biomaterials are integral to advancing drug delivery, offering diverse applications that go beyond conventional uses. They have a transformative impact on cancer treatment, where biomaterial-based nanoparticles and liposomes can precisely deliver chemotherapeutic agents to malignant cells, minimizing side effects. In managing cardiovascular diseases, biomaterial-coated drug-eluting stents release medications locally, reducing restenosis risks. Overcoming the blood–brain barrier, biomaterials aid in targeted drug delivery for neurodegenerative diseases like Alzheimer’s and Parkinson’s. In vaccine development, biomaterials optimize immune responses by enabling controlled antigen release. Additionally, biomaterial scaffolds in tissue engineering facilitate cell growth and differentiation, promising advancements in artificial skin, cartilage repair, and functional organs. In ocular conditions, sustained release drug-delivery systems enhance the efficacy of treatments for diseases like glaucoma and macular degeneration. Overall, biomaterials revolutionized the pain management through the development of localized, sustained-release drug-delivery systems [234,235,236]. These systems can be implanted or injected near the source of pain, ensuring that analgesic medications are delivered directly to the affected area, reducing the potential for systemic side effects and dependency.

Despite the numerous benefits that biomaterials bring to drug delivery, such as enhanced drug stability, minimized side effects, and precise administration, persistent challenges remain. These challenges involve potential toxicity considerations, intricate manufacturing processes, and the requirement for stringent regulatory approval procedures [237,238,239]. Moreover, the era of personalized medicine and patient-specific drug-delivery systems introduces unique complexities and considerations, mandating ongoing research and innovation in the field. The versatile nature of biomaterials continues to drive advancements in drug delivery, paving the way for more effective, efficient, and patient-centered healthcare solutions.

## 5. Conclusions

Recent biomaterial breakthroughs in drug delivery herald a new era of precision medicine, with smart biomaterials that are responsive to physiological cues for on-demand drug release [212,240,241]. Innovations in nanotechnology and gene therapy further enhance personalized drug delivery, transforming healthcare. Biomaterials play a large role in tailoring treatments based on individual genetic profiles and disease characteristics, optimizing outcomes and reducing adverse reactions. They facilitate access to once-inaccessible areas like the brain, promising significant improvements in conditions such as Alzheimer’s and specific cancers. Scaffold materials, with intricate microarchitecture, guide tissue regeneration, while hydrogels provide a supportive environment for stem cell growth and differentiation [242]. Real-time monitoring and feedback systems in biomaterial-based drug delivery dynamically adjust drug-release rates, ensuring patients receive precise doses when needed. The evolving landscape necessitates improved regulatory frameworks for seamless integration into mainstream healthcare. Biomaterials precisely control drug release, enhancing efficacy and minimizing side effects. Ongoing research promises groundbreaking developments, revolutionizing healthcare and defining 21st century medicine with personalized, targeted drug-delivery solutions. These innovations redefine healthcare possibilities, offering tailored treatments for individual needs with unparalleled accuracy, positioning biomaterials as a beacon of hope for patients and a driving force in medical practice evolution.

## Figures and Tables

**Table 1:** A list of biomaterials used for drug-delivery systems.

Biomaterial	Description	Applications	Ref.
Poly(lactic-co-glycolic acid) (PLGA)	Biodegradable copolymer used for sustained drug release	Microspheres, nanoparticles, implants	[84]
Liposomes	Spherical lipid vesicles that can encapsulate hydrophobic and hydrophilic drugs	Targeted drug delivery, gene therapy	[85]
Alginate	Natural polysaccharide derived from algae, forms hydrogels	Controlled drug release, wound healing	[86]
Chitosan	Biopolymer derived from chitin, forms hydrogels	Oral drug delivery, wound dressings	[87]
Hyaluronic Acid (HA)	Natural component of the extracellular matrix	Ophthalmic, joint injections, skin creams	[88]
Polyethylene Glycol (PEG)	Synthetic polymer used to improve drug solubility	Nanoparticles, conjugates	[89]
Cyclodextrins	Cyclic oligosaccharides used for drug encapsulation	Increasing drug solubility	[90]
Dendrimers	Highly branched macromolecules with precise structures	Targeted drug delivery, gene therapy	[91]
Polylactic Acid (PLA)	Biodegradable polymer	Nanoparticles, implants	[92]
Polymeric Micelles	Self-assembling structures formed by amphiphilic block copolymers	Solubilization and delivery of hydrophobic drugs	[93]

The choice of biomaterial depends on the specific drug and delivery requirements. More detailed information, such as release kinetics, biocompatibility, and specific drug-delivery applications, would be necessary for a comprehensive analysis. Additionally, the field of drug delivery is continually evolving, and new biomaterials and technologies are being developed regularly.

**Table 2:** A list of biomaterials used for tissue engineering.

Biomaterial	Description	Applications	Ref.
Collagen	Major structural protein in the extracellular matrix	Skin, bone, cartilage, nerve, and dental tissue engineering	[97]
Hyaluronic Acid (HA)	Natural component of connective tissues	Ophthalmic, osteoarthritis, and wound healing	[98]
Poly(lactic-co-glycolic acid) (PLGA)	Biodegradable copolymer	Scaffold for various tissues, such as bone, cartilage, and nerves	[99]
Chitosan	Biopolymer derived from chitin	Skin, cartilage, bone, and vascular tissue engineering	[100]
Decellularized Extracellular Matrix (ECM)	Natural tissue matrix with cells removed	Various tissues, including heart, liver, and kidney	[101]
Gelatin	Derived from collagen, biocompatible	Soft tissue engineering, cell encapsulation	[102]
Silk	Natural protein fiber	Nerve regeneration, skin, and bone tissue engineering	[103]
Alginate	Derived from seaweed, forms hydrogels	Encapsulation and 3D printing of cells and tissues	[104]
Polycaprolactone (PCL)	Biodegradable synthetic polymer	Bone and cartilage tissue engineering	[105]
Polyglycolic Acid (PGA)	Biodegradable synthetic polymer	Scaffold for various tissues, including bone and cartilage	[106]
Polyethylene Glycol (PEG)	Biocompatible synthetic polymer	Cell encapsulation, vascular tissue engineering	[107]
Calcium Phosphates	Bioceramics, such as hydroxyapatite and tricalcium phosphate	Bone and dental tissue engineering	[108]

The choice of biomaterial depends on the specific tissue being engineered, the desired properties, and the intended clinical application. Tissue engineering is a rapidly evolving field, and researchers continually explore new biomaterials and technologies to enhance tissue regeneration and transplantation.

**Table 3:** A list of biomaterials used for implantable devices.

Biomaterial	Description	Properties/Advantages	Ref.
Titanium and Titanium Alloys	Lightweight, strong, corrosion-resistant metals	Orthopedic implants (joint replacements, bone plates)	[109]
Stainless Steel	Durable, corrosion-resistant steel alloy	Stents, orthopedic implants	[110]
Cobalt-Chromium Alloys	High-strength alloys with excellent wear resistance	Orthopedic implants, heart valves	[111]
Polyethylene	Durable and biocompatible polymer	Joint replacements (as bearing surfaces)	[112]
Silicone	Biocompatible elastomer	Breast implants, catheters	[113]
Polymethyl Methacrylate (PMMA)	Biocompatible thermoplastic polymer	Bone cement for joint replacements	[114]
Polyurethane	Flexible and biocompatible polymer	Heart valves, catheters, pacemaker leads	[115]
Polyetheretherketone (PEEK)	Biocompatible thermoplastic polymer	Spinal implants, dental devices	[116]
Zirconia	Bioceramic with excellent mechanical properties	Dental implants, hip replacements	[117]
Poly-Lactic Acid (PLA)	Biodegradable polymer	Temporary implants and drug-eluting devices	[118]
Hydrogels	Water-absorbent materials with tunable properties	Drug delivery, tissue scaffolds	[119]
Nitinol (Nickel-Titanium)	Shape memory alloy	Stents, guidewires, and orthopedic devices	[120]
Tantalum	Biocompatible metal with high corrosion resistance	Orthopedic implants and bone screws	[121]

Also in this case, the choice of biomaterial depends on the specific device, its intended function, and its compatibility with the surrounding tissues. Innovations in materials science continue to expand the range of biomaterials available for implantable medical devices.

**Table 4:** A list of biomaterials used for diagnostic tools.

Biomaterial	Application	Properties/Advantages	Ref.
Silicon	Microfluidic chips, sensors	High thermal conductivity, compatibility with electronics	[122]
Glass	Microscope slides, microfluidics	Transparency, chemical resistance, biocompatibility	[123]
Polydimethylsiloxane (PDMS)	Microfluidic devices	Transparency, flexibility, ease of fabrication	[124]
Polystyrene (PS)	Cell culture dishes, multi-well plates	Biocompatibility, rigidity	[125]
Polyethylene (PE)	Disposable pipettes, tubes	Low cost, chemical resistance, ease of molding	[126]
Polypropylene (PP)	Disposable microplates	Chemical resistance, thermal stability	[127]
Polycarbonate (PC)	Microplates, labware	Clarity, durability, heat resistance	[128]
Polyurethane (PU)	Catheters, tubing	Flexibility, durability, biocompatibility	[129]
Gold	Biosensors, nanoparticles	High surface area, conductive properties, bioconjugation	[130]
Nitrocellulose	Immunoassays, lateral flow assays	Rapid capillary flow, protein binding capacity	[131]
Hydrogels	Drug delivery, biosensors	Water absorption, biocompatibility, tunable properties	[132]
Paper	Lateral flow assays, diagnostic cards	Low cost, portability, capillary flow	[133]
Biodegradable polymers	Controlled drug release	Biocompatibility, controlled degradation	[134]
Magnetic nanoparticles *	Magnetic resonance imaging (MRI)	Magnetic properties for contrast enhancement	[135]

* In medical imaging, particularly techniques like magnetic resonance imaging (MRI), contrast agents are often employed to enhance the visibility of specific tissues or structures. These contrast agents, which possess magnetic properties, are introduced into the body to create a clearer distinction between different tissues, leading to improved imaging results.

**Table 5:** A list of biomaterials used for regenerative medicine.

Biomaterial	Application	Properties/Advantages	Refs.
Collagen	Tissue engineering, wound healing	Natural component of extracellular matrix, biocompatible	[136]
Hyaluronic Acid	Dermal fillers, tissue scaffolds	High water-binding capacity, lubrication, biocompatible	[137]
Chitosan	Scaffolds, drug delivery	Biodegradable, biocompatible, antimicrobial properties	[138]
Alginate	Cell encapsulation, tissue engineering	Gel-forming, biocompatible, ease of shaping	[139]
Poly(lactic-co-glycolic acid)	Drug delivery, scaffolds	Biodegradable, tunable degradation rates, biocompatible	[140]
Decellularized Tissues	Organ and tissue transplantation	Retains natural extracellular matrix, minimizes immune response	[141]
Hydroxyapatite	Bone grafts, dental applications	Similar composition to bone mineral, osteoconductive	[138]
Demineralized Bone Matrix (DBM)	Bone regeneration	Osteoinductive properties, supports new bone growth	[142]
Polycaprolactone	Scaffolds, tissue engineering	Biodegradable, mechanical strength	[143]
Fibrin	Wound healing, tissue engineering	Natural clotting protein, supports cell migration	[144]
Silk Fibroin	Tissue scaffolds, drug delivery	Biocompatible, mechanical strength, tunable degradation	[145]
PEG	Hydrogels, drug delivery	Biocompatible, tunable properties	[146]
Stem Cells	Regeneration of various tissues	Pluripotent or multipotent cells for tissue repair	[147]
ECM	Tissue engineering, wound healing	Retains tissue-specific structural cues, promotes cell adhesion	[148]

Regenerative medicine involves a wide range of biomaterials tailored for specific applications, and this table provides a general overview of some commonly used materials in the field.

**Table 6:** A list of biomaterials used for drug screening and research.

Biomaterial	Application	Properties/Advantages	Refs.
HEK 293 Cells	Protein expression, drug target validation.	High transfection efficiency, widely used.	[149]
MCF-7 Cells	Breast cancer research, drug screening.	Representative for breast cancer.	[150]
A549 Cells	Lung cancer and respiratory disease drug studies.	Relevant for lung cancer.	[151]
HUVEC (Human Umbilical Vein Endothelial Cells)	Vascular and cardiovascular drug research.	Mimic human vascular conditions.	[152]
CaCO-2 Cells	Drug transport and oral absorption studies.	Representative of the intestinal barrier.	[153]
iPSCs (Induced Pluripotent Stem Cells)	Disease modeling and personalized medicine.	Generated from patient cells.	[154]
3D Cell Models	Physiologically relevant cell behavior studies.	Recapitulate tissue-like environments.	[155]
Hepatocytes	Liver metabolism and toxicity studies.	Essential for liver drug metabolism research.	[156]
THP-1 Cells	Inflammation and immune response drug research.	Immunology and drug screening.	[157]
Tumor Xenograft Models	Replicate human cancer for in vivo drug testing.	Mimic human cancer conditions for testing.	[158]
Zebrafish Models	High-throughput screening and developmental studies.	Transparent embryos for live imaging.	[159]
Human Tissue Microarrays	Tissue sample analysis and biomarker identification.	Efficient analysis of multiple samples.	[160]
Chitosan-Based Hydrogels	Tissue engineering and controlled drug delivery.	Biocompatible, versatile scaffold.	[161]
PLGA Nanoparticles	Drug encapsulation and targeted drug delivery.	Controlled release.	[162]
Monoclonal Antibodies	Biologic drug development and immunotherapy.	High specificity and therapeutic potential.	[163]
Peptide Nucleic Acids	Genetic research, molecular biology, and gene therapy.	Sequence-specific hybridization.	[164]

These specific biomaterials offer distinct advantages in drug screening and research, enhancing the ability of researchers to conduct experiments, develop drugs, and understand disease mechanisms more effectively.

**Table 7:** A list of biomaterials used for bioimaging.

Biomaterial	Application	Properties/Advantages	References
Quantum Dots	Fluorescent labeling for cellular and molecular imaging.	High brightness, tunable emission, and photostability.	[165]
Fluorescent Proteins	Tagging proteins and tracking cellular processes.	Genetically encoded, non-invasive imaging.	[166]
Gold Nanoparticles	Contrast agents for electron microscopy and optical imaging.	Excellent light scattering and high contrast.	[165]
Iron Oxide Nanoparticles	Magnetic resonance imaging (MRI) contrast agents.	High relaxivity for sensitive MRI detection.	[167]
Upconversion Nanoparticles	Multispectral imaging and deep tissue imaging.	Low autofluorescence and minimal photodamage.	[168]
Carbon Nanotubes	Optical imaging and drug-delivery carriers.	Strong absorbance in the near-infrared region.	[169]
Fluorescent Dyes	Versatile for various bioimaging techniques.	Bright fluorescence and broad color range.	[170]
Liposomes	Drug delivery and encapsulation of imaging agents.	Easy modification and controlled drug release.	[171]
Magnetic Nanoparticles	Magnetic resonance imaging (MRI) contrast agents.	Safe and non-toxic for in vivo imaging.	[172]
Silica Nanoparticles	Labeling and tracking cellular events.	High stability and easy surface functionalization.	[173]
Gold Nanorods	Imaging and photothermal therapy applications.	Strong absorbance in the near-infrared region.	[174]
Biosensors	Detect and quantify specific biomolecules in real-time.	High sensitivity and specificity.	[175]
Nanodiamonds	Multimodal imaging, including MRI and fluorescent imaging.	Stable and long-lasting fluorescence.	[176]
Optical Coherence Tomography (OCT)	Enhance depth-resolved imaging in ophthalmology.	Real-time imaging and non-invasive.	[177]
Peptide Nanotubes	Cellular imaging and drug delivery.	Biocompatibility and ease of functionalization.	[178]

These specific biomaterials offer distinct advantages in bioimaging, allowing researchers and clinicians to visualize and understand biological processes, diseases, and structures with precision and efficiency.

**Table 8:** A list of biomaterials used for vaccine development.

Biomaterial	Application	Properties/Advantages	References
Adjuvants (Aluminum salts)	Enhance immune response and vaccine efficacy.	Proven safety and effectiveness in many vaccines.	[179]
Virus-like Particles (VLPs)	Mimic viral structures to stimulate the immune system.	Safer than using live or inactivated viruses.	[180]
Recombinant Proteins	Present antigens to induce specific immune responses.	Highly purified and well-defined antigens.	[181]
DNA Vaccines	Deliver genetic material encoding antigens for immune response.	Easy production and modification.	[182]
mRNA Vaccines	Use synthetic mRNA to instruct cells to produce antigens.	Rapid development and potential for personalized vaccines.	[183]
Viral Vector Vaccines	Modified viruses deliver antigens to trigger immune response.	Strong and long-lasting immune responses.	[184]
Live Attenuated Vaccines	Use weakened forms of pathogens for immune stimulation.	Often provide robust and long-lasting immunity.	[185]
Inactivated Vaccines	Use killed pathogens to induce an immune response.	Safer than live vaccines but still provide protection.	[186]
Nanoparticles (e.g., liposomes)	Deliver antigens and adjuvants to enhance vaccine performance.	Improved stability and immune response.	[187]
Protein Subunit Vaccines	Use purified pieces of pathogens as antigens.	Safe and well-tolerated, no risk of causing disease.	[188]
Virus Vectored Subunit Vaccines	Combine virus vectors with subunit antigens for strong immune response.	Induce both cellular and humoral immune responses.	[189]
Polysaccharide Conjugate Vaccines	Link bacterial polysaccharides to proteins for better immune recognition.	Effective against bacterial diseases.	[190]
Lipid Nanoparticles (LNPs)	Deliver mRNA and other vaccine components efficiently.	Facilitate cellular uptake and mRNA translation.	[191]
Cell-Based Vaccines	Use cultured cells to produce antigens for vaccine development.	Scalable and adaptable for various pathogens.	[192]
Adenovirus-based Vaccines	Utilize adenoviruses to deliver genetic material for antigen production.	Strong immune responses, particularly against viral diseases.	[193]

These biomaterials offer distinct advantages in vaccine development, while preventing a wide range of infectious diseases.

## Data Availability

Data is contained within the article.

## Abbreviation List

Poly(lactic-co-glycolic acid) (PLGA), Hyaluronic Acid (HA), Polyethylene Glycol (PEG), Extracellular Matrix (ECM), Polycaprolactone (PCL), Polylactic Acid (PLA), Polyglycolic Acid (PGA), Polymethyl Methacrylate (PMMA), Polyetheretherketone (PEEK), Poly-Lactic Acid (PLA), Polydimethylsiloxane (PDMS), Polystyrene (PS), Polyethylene (PE), Polypropylene (PP), Polycarbonate (PC), Polyurethane (PU), Magnetic resonance imaging (MRI), Demineralized Bone Matrix (DBM), iPSCs (Induced Pluripotent Stem Cells), Optical Coherence Tomography (OCT), Virus-like Particles (VLPs), Lipid Nanoparticles (LNPs), Polyethylene Oxide (PEO), Polyvinyl Alcohol (PVA), and Polybutylene Adipate Terephthalate (PBAT).
